# Relationships Between Sleep Quality, Perceived Stress, and Premenstrual Syndrome Among Medical and Nursing Students: A Cross-Sectional Study in Palestine

**DOI:** 10.3390/healthcare14091168

**Published:** 2026-04-27

**Authors:** Malak Abu Khashan, Shahd Aldarak, Marwa Zahdeh, Ayah Alawneh, Nada Abo Dyak, Shahd Qazae, Shahd Ghanem, Mohammad N. S. Al-Mohtaseb, Hadeel Ayesh, Abdallah Alwawi, Azzam Zrineh

**Affiliations:** 1 Faculty of Medicine, Al-Quds University, Jerusalem P.O. Box 3523, Palestine; malak.khashan16@gmail.com (M.A.K.); marwarafat41@gmail.com (M.Z.); azamshahd795@gmail.com (S.Q.); hadeelayesh1999@gmail.com (H.A.); azzamzrineh@gmail.com (A.Z.); 2 Department of Medicine, Faculty of Medicine and Allied Medical Sciences, An-Najah National University, Nablus P.O. Box 7, Palestine; shahddarak7@gmail.com (S.A.); ayakamel2810@gmail.com (A.A.); nadaabodyak@gmail.com (N.A.D.); 3 Faculty of Medicine, Mansoura University, Mansoura 35516, Egypt; ghanemshahd4@gmail.com; 4 Alexandria Faculty of Medicine, Alexandria University, Alexandria 21526, Egypt; mohammad.soliman1801@alexmed.edu.eg; 5 Faculty of Health Professions, Al-Quds University, Jerusalem P.O. Box 3523, Palestine

**Keywords:** healthcare education, medical students, nursing students, perceived stress, premenstrual syndrome, sleep quality

## Abstract

Background: Premenstrual syndrome (PMS) significantly impacts daily functioning among women of reproductive age. Medical and nursing students face exceptional stressors that may exacerbate PMS, yet the interrelationships between sleep quality, perceived stress, and PMS remain understudied in Middle Eastern contexts. This study aimed to investigate the relationships between sleep quality, perceived stress, and self-reported PMS symptoms among Palestinian female medical and nursing students, to assess their prevalence and severity, and to examine variations across sociodemographic and lifestyle factors. Methods: This cross-sectional correlational study recruited 708 female medical and nursing students from three Palestinian universities. Validated Arabic instruments assessed sleep quality (PSQI), perceived stress (PSS-10), and PMS symptoms (A-PMSS). Analyses included Spearman’s correlations, non-parametric tests (Mann–Whitney U Test, Kruskal–Wallis Test), and multivariable linear regression. Results: Nearly all participants (98%) reported some degree of self-reported PMS symptoms at any severity level, with 76% experiencing moderate-to-severe symptoms. Poor sleep quality (PSQI > 5) affected 62.1%, while 94.5% reported moderate-to-high perceived stress. Significant positive correlations were identified between PSQI and PMS (rho = 0.295, *p* < 0.001) and between PSS-10 and PMS (rho = 0.483, *p* < 0.001). Multivariable regression revealed that perceived stress and sleep quality were significant independent predictors of PMS severity after adjusting for covariates, with the overall model explaining 27.5% of variance in PMS scores. Conclusions: This study reveals a substantial burden of PMS, poor sleep quality, and stress among Palestinian female medical and nursing students. Perceived stress and sleep quality were independently associated with PMS severity. These findings highlight need for integrated wellness programs in healthcare education for Palestinian students.

## 1. Introduction

Premenstrual syndrome (PMS) is a complex cyclical disorder that involves a range of physical and psychological symptoms that arise during the luteal phase of the menstrual cycle, often causing notable distress and functional limitations. These symptoms typically resolve within a few days after menstruation begins. A meta-analysis of studies across different countries estimated a pooled prevalence of 47.8% (95% CI: 32.6 to 62.9) for PMS [1], though reported rates vary widely depending on diagnostic criteria, assessment tools, and study populations. It is important to note that epidemiological studies assessing PMS through self-report questionnaires measure self-reported symptom presence and severity, which differs from formal clinical diagnosis requiring prospective symptom tracking as per DSM-5 criteria.

Common PMS symptoms include appetite changes, weight gain, abdominal and back pain (including lower-back pain), headaches, breast swelling and tenderness, nausea, constipation, fatigue, irritability, restlessness, anxiety, anger, mood swings, and episodes of crying [2].

The etiology of PMS is complex and not completely understood. Hormonal fluctuations, especially in estrogen and progesterone levels throughout the menstrual cycle, are thought to play a central role. These hormonal changes can affect neurotransmitter systems, particularly serotonin and gamma-aminobutyric acid (GABA), contributing to PMS manifestations [3]. Furthermore, genetic factors, lifestyle influences, and psychosocial stressors can affect the development and severity of PMS [3,4,5].

Medical and nursing students represent a unique population who usually face exceptional academic and clinical stressors that may exacerbate PMS symptoms. The demanding nature of healthcare education, characterized by intensive schedules, clinical responsibilities, sleep deprivation, and exposure to emotionally challenging situations, creates an environment where stress-related mental health issues are highly prevalent [6]. Previous research has shown that healthcare students, particularly those in medical and nursing programs, commonly experience high levels of sleep disturbances alongside mental health issues, including depression, anxiety, and stress [7,8,9,10,11].

Sleep disturbances are recognized as significant factors in women’s reproductive health. Research indicates that university students experience high rates of poor sleep quality, with a recent global systematic review and meta-analysis reporting an estimated pooled prevalence of 55.64% among medical students [12]. Poor sleep quality has been associated with PMS symptoms [13,14], while PMS symptoms themselves can further impair sleep quality, creating a potential cyclical pattern. However, the direction of this association remains unclear, and it is uncertain whether menstrual issues lead to poor sleep or if poor sleep contributes to menstrual issues [13]. Poor sleep has been associated not only with adverse health outcomes but also with diminished academic performance and quality of school life among students [15].

Caffeine consumption, on the other hand, is particularly relevant in healthcare student populations, as students may use caffeine to cope with sleep deprivation during demanding academic and clinical schedules; however, its excessive use has been associated with poor sleep quality and elevated stress in some studies [16,17,18,19].

Stress has emerged as a critical factor in PMS, with evidence suggesting a complex bidirectional relationship with premenstrual symptoms [20]. From a biological perspective, women with PMS demonstrate abnormal stress reactivity patterns characterized by dysregulation of the hypothalamic–pituitary–adrenal (HPA) axis, where stress-induced cortisol disrupts the hypothalamic–pituitary–ovarian (HPO) axis, while hormonal fluctuations during the menstrual cycle heighten sensitivity to stress [20]. There is significant comorbidity between PMS and stress-related disorders such as PTSD, with trauma history, particularly sexual trauma, strongly associated with more severe PMS [20]. PMS itself may function as a chronic stressor, creating a negative feedback loop where premenstrual symptoms induce stress, which in turn exacerbates symptom severity [20].

In Palestine, female students comprise a significant proportion of medical and nursing school enrollments, yet limited research has examined the burden of premenstrual symptoms and their relationship with sleep quality and stress. Abu Alwafa et al. [21] conducted a pioneering study documenting an extremely high rate of self-reported PMS symptoms among female Palestinian university students, providing important baseline data.

The Palestinian context presents distinctive challenges for student health and well-being. Chronic exposure to socio-political stressors, including military checkpoints restricting movement to educational institutions, economic instability, recurrent conflict-related disruptions, and limited access to mental health services, may directly elevate perceived stress levels and disrupt sleep patterns, which are two of the key variables examined in this study. Additionally, cultural norms around discussing menstrual health may limit help-seeking behaviors for reproductive health concerns.

Taken together, the existing literature suggests that sleep disturbances, perceived stress, and PMS may be interconnected through shared neurobiological pathways. Dysregulation of the hypothalamic–pituitary–adrenal (HPA) axis under chronic stress can disrupt the hypothalamic–pituitary–ovarian (HPO) axis, while sleep disruption and hormonal fluctuations may further impair serotonergic and melatoninergic function. Both systems are implicated in mood regulation and premenstrual symptomatology [20,22,23,24]. We therefore hypothesized that sleep quality and perceived stress would each be significantly and independently associated with PMS symptom severity among medical and nursing students in Palestine, a population facing both academic and contextual stressors.

This study addressed the following research questions: (a) What is the prevalence and severity of self-reported PMS symptoms among female medical and nursing students in Palestine? (b) What are the sleep quality profiles and perceived stress levels among this population? (c) Are sleep quality and perceived stress significantly correlated with PMS symptom severity? (d) Do sociodemographic and lifestyle factors significantly influence variations in PMS severity, sleep quality, and perceived stress levels?

## 2. Methods

### 2.1. Study Design and Settings

This was a cross-sectional correlational study conducted among female medical and nursing students enrolled in three major Palestinian universities: Al-Quds University (Jerusalem), An-Najah National University (Nablus), and Hebron University (Hebron). These institutions are among the largest healthcare education providers in Palestine, and they are serving students from diverse geographic and socioeconomic backgrounds. Data collection in our study took place between 8 October and 15 November 2025, through an online survey distributed via social media platforms. The reporting of this study was informed by the Strengthening the Reporting of Observational Studies in Epidemiology (STROBE) guidelines [25] (Table S1).

### 2.2. Eligibility Criteria

Eligibility criteria included participants meeting the following inclusion criteria: Female medical or nursing students currently enrolled in one of the three participating universities; students in any academic year: medical students (1st through 6th year) and nursing students (1st through 4th year); regular menstrual cycles for the past 6 months; and willingness to provide informed consent.

Exclusion criteria included the following: students diagnosed with medical conditions affecting sleep or menstrual cycle; current use of hormonal contraceptives or hormonal contraceptive use within the past 6 months; current pregnancy or breastfeeding; and incomplete questionnaire responses (defined as any missing items across the three standardized instruments: PSQI, PSS-10, or A-PMSS).

### 2.3. Sample Size Calculation and Sampling Method

A priori sample size calculation for correlation analysis (power = 0.95, α = 0.05, effect size = 0.3) yielded a minimum required sample of 140 participants using StatsDirect: Version 4.0.4 software. The final sample of 708 participants substantially exceeds this requirement, providing ample statistical power for all planned analyses, including subgroup comparisons. We used convenience sampling via online survey distribution through social media platforms due to accessibility constraints in the Palestinian context.

### 2.4. Data Collection Instruments

We collected data through a self-administered online questionnaire distributed via Google Forms (Google LLC, Mountain View, CA, USA) through social media platforms. The questionnaire we used comprised four sections: the first covered sociodemographic, clinical, and lifestyle characteristics, the second included the Arabic Pittsburgh Sleep Quality Index (PSQI), the third included the Arabic Perceived Stress Scale (PSS-10), and the fourth was the Arabic Premenstrual Syndrome Scale (A-PMSS).

#### 2.4.1. Sociodemographic, Clinical, and Lifestyle Characteristics

Participants provided information on variables such as age, marital status, living arrangements, residence, university affiliation, major (medicine vs. nursing), academic year, menstrual cycle characteristics, contraceptive use, prior diagnoses, and some lifestyle factors such as caffeine consumption, physical activity levels, and smoking status.

#### 2.4.2. Pittsburgh Sleep Quality Index (PSQI)

Sleep quality was measured using the validated Arabic version of the Pittsburgh Sleep Quality Index (PSQI) [26]. PSQI is a widely used 19-item self-report questionnaire assessing sleep quality, with a global score ranging from 0 to 21, with higher scores indicating poorer sleep quality. A global PSQI score > 5 was used as an established cut-off of poor sleep quality [27]. The Arabic PSQI has been extensively used and validated in Arab populations, including medical students [28,29,30]. In the present sample, the PSQI demonstrated good internal consistency (Cronbach’s α = 0.835).

#### 2.4.3. Perceived Stress Scale (PSS-10)

Perceived stress was assessed using the Arabic version of the 10-item Perceived Stress Scale (PSS-10), validated by Ali et al. [31]. The PSS-10 measures the degree to which situations in one’s life are appraised as stressful. Respondents rate each item on a 5-point Likert scale (0 = never, 1 = almost never, 2 = sometimes, 3 = fairly often, 4 = very often). Four items (4, 5, 7, and 8) are reverse-scored. The total PSS-10 score is calculated by summing all item responses after reverse-scoring the positively worded items, with higher scores indicating greater perceived stress. PSS-10 total score ranges from 0 to 40. Accordingly, stress levels are categorized as: low (scores 0–13), moderate (scores 14–26), and high (scores 27–40). The Arabic PSS-10 has been previously used and validated within Arabic-speaking populations in different contexts [32,33,34]. In the present sample, the PSS-10 demonstrated acceptable internal consistency (Cronbach’s α = 0.765).

#### 2.4.4. Arabic Premenstrual Syndrome Scale (A-PMSS)

PMS was assessed using the Arabic Premenstrual Syndrome Scale (A-PMSS), a 23-item instrument developed by Algahtani and Jahrami [35] according to DSM-IV-TR criteria, specifically designed and validated for Arabic-speaking populations. The A-PMSS evaluates symptoms experienced during the week before menstruation over the past 3 months, organized into three domains. The psychological symptoms domain includes items 1–10 and 16–17, assessing depressed mood, hopelessness, feeling guilty, anxiety/worry, affective lability, increased sensitivity toward others, feeling angry, easily irritated/agitated, lack of interest, difficulty concentrating, loss of control, and feeling overwhelmed. The physical symptoms domain encompasses items 11–15 and 18–22, covering lethargy/fatigue/decreased energy, increased appetite, craving certain foods, hypersomnia, insomnia, breast tenderness, breast engorgement or weight gain, headache, muscle/joint/back pain, and acne. The functional impairment domain, represented by item 23, includes three subcategories examining interference with relationships, work/school performance, and daily routine.

Each item is rated on a 4-point Likert scale, and the scores for each domain were calculated as the average of constituent items. The total PMS score was computed as the average across all items, providing an overall severity measure (ranging from 0 to 3). Participants were subsequently categorized into four severity groups: None (score = 0), Mild (score > 0 to ≤1), Moderate (score > 1 to ≤2), and Severe (score > 2). Importantly, the A-PMSS tool has been used in a previous study by Abu Alwafa et al. [21] among Palestinian female university students, confirming its validity and reliability for use in our study. In the present sample, the A-PMSS demonstrated excellent internal consistency for the total scale (Cronbach’s α = 0.953) and the psychological domain (α = 0.942), and good internal consistency for the physical domain (α = 0.867) and the functional impairment domain (α = 0.815).

### 2.5. Statistical Analysis

Data were analyzed using Jamovi software: Version 2.3.28. Descriptive statistics were computed for all variables; continuous variables were reported as the median and interquartile range (IQR), as well as the mean and standard deviation (SD) for the summary statistics of PMS, sleep quality, and perceived stress, while categorical variables were reported as frequencies and percentages. Normality was assessed using the Shapiro–Wilk test, which confirmed significant departures from normality for all major continuous study variables (all *p* < 0.001), which was corroborated by visual inspection of histograms and Q-Q plots. Given the substantial deviations from normality, non-parametric statistical methods were employed for bivariate analyses. Spearman’s rank correlation coefficient (rho) was used to examine bivariate relationships between continuous study variables. The Mann–Whitney U test was used to compare distributions between two independent groups, and the Kruskal–Wallis test for comparisons across three or more groups. Additionally, multivariable linear regression analysis was conducted to examine independent predictors of PMS severity, with total PMS score as the dependent variable and PSQI total score, PSS-10 total score, age, academic year, caffeine consumption, and physical activity level as independent variables. Multicollinearity was assessed using variance inflation factors (VIF < 5 considered acceptable), and the Durbin–Watson statistic was used to test for autocorrelation of residuals. Incomplete questionnaire responses were excluded, and statistical significance was set at *p* < 0.05 for all analyses.

## 3. Results

### 3.1. Participants’ Characteristics

A total of 881 responses were received, of which 173 (19.6%) were excluded for meeting at least one predefined exclusion criterion, yielding a final analytical sample of 708 participants. The median age of the sample was 21 years (IQR = 3, range: 18–32 years). The majority were single (96.9%, n = 686), with only 3% (n = 21) married and 0.1% (n = 1) divorced. The sample was distributed across three universities: An-Najah University (44.8%, n = 317), Al-Quds University (30.2%, n = 214), and Hebron University (25%, n = 177). Medical students comprised 59.2% (n = 419) of the sample, with nursing students representing 40.8% (n = 289). The median age of menarche was 13 years (IQR = 2). Regarding self-reported menstrual pain severity, 4.2% (n = 30) reported no pain, 26.6% (n = 188) mild pain, 44.2% (n = 313) moderate pain, and 25% (n = 177) severe pain. Regarding lifestyle factors, caffeine consumption was reported as never by 7.3% (n = 52), rarely by 17.8% (n = 126), sometimes by 22.6% (n = 160), daily by 42.5% (n = 301), and excessive consumption by 9.7% (n = 69). Smoking was reported by 3.4% (n = 24) of participants. Detailed sociodemographic, clinical, and lifestyle characteristics are presented in Table 1 and Table 2.

### 3.2. Prevalence and Severity of Self-Reported PMS Symptoms

Only 2% (n = 14) of participants reported no PMS symptoms, indicating that 98% of participants reported at least one PMS symptom at any severity level. Among those experiencing symptoms, 22% (n = 156) reported mild symptoms, while the majority experienced moderate to severe symptoms, with 47% (n = 333) reporting moderate symptoms and 29% (n = 205) reporting severe symptoms. The combined proportion reporting moderate-to-severe PMS symptoms was 76% (n = 538). 

Domain scores revealed that psychological symptoms (median = 1.75, IQR = 1.17) were slightly higher than physical symptoms (median = 1.50, IQR = 1). Functional impairment showed a median score of 1.33 (IQR = 1.33). The overall median total PMS score for the sample was 1.6 (IQR = 1).

### 3.3. Individual PMS Symptom Prevalence

The most frequently reported PMS symptoms were predominantly psychological. Lethargy/fatigue/decreased energy was the most common symptom, reported by 92.7% (n = 656) of participants at any severity level, followed by anxiety/worry (90.8%, n = 643), depressed mood (90.6%, n = 642), a lack of interest (90.2%, n = 638), and feeling angry (89.5%, n = 634). Among physical symptoms, muscle/joint/back pain was reported by 88.9% (n = 630) of participants. Increased sensitivity toward others (88.3%, n = 625), difficulty concentrating (87.6%, n = 620), mood swings (86.9%, n = 616), and feeling overwhelmed (86.8%, n = 614) were also highly prevalent. Prevalence percentages represent participants experiencing the symptom at any severity level (mild, moderate, or severe) during the week before menstruation.

### 3.4. Sleep Quality and Perceived Stress

Sleep quality assessment revealed that the median global PSQI score was 7 (IQR = 5), exceeding the PSQI cutoff of 5 for poor sleep quality. When dichotomized using this threshold, 62.1% (n = 440) of participants demonstrated poor sleep quality (PSQI > 5), while 37.9% (n = 268) reported good sleep quality (PSQI ≤ 5). Perceived stress assessment using the PSS-10 yielded a median score of 20.5 (IQR = 9), falling within the moderate stress category. Distribution across stress categories showed: low stress 5.5% (n = 39), moderate stress 72.9% (n = 516), and high stress 21.6% (n = 153). Notably, 94.5% of participants experienced moderate to high stress levels, indicating substantial stress burden in this population. Table 3 presents prevalence, severity, and summary statistics for PMS, sleep quality, and perceived stress.

### 3.5. Correlations Between Sleep Quality, Perceived Stress, and PMS

**Figure 1:**
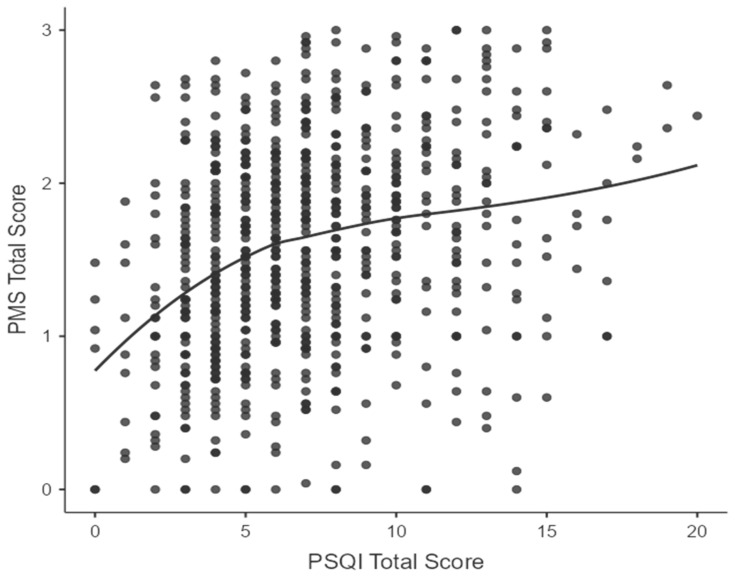
A scatter plot showing the correlation between PSQI total score and total PMS score.

Spearman’s rank correlation analyses revealed significant positive correlations between all primary study variables (see Table 4). Sleep quality (PSQI score) demonstrated a statistically significant positive correlation with total PMS score (rho = 0.295, *p* < 0.001), indicating that poorer sleep quality was associated with more severe PMS symptoms. (See Figure 1). When examined by PMS domains, PSQI scores correlated significantly with psychological symptoms (rho = 0.248, *p* < 0.001), physical symptoms (rho = 0.278, *p* < 0.001), and functional impairment (rho = 0.317, *p* < 0.001).

**Figure 2:**
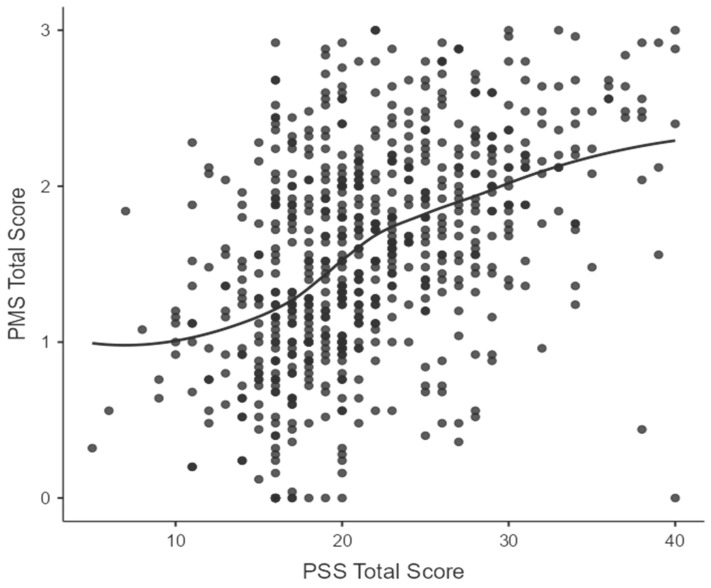
A scatter plot showing the correlation between PSS total score and total PMS score.

Perceived stress (PSS-10 score) showed stronger correlations with PMS compared to sleep quality (PSQI) across all domains. A moderate positive correlation was observed between perceived stress (PSS-10) and total PMS score (rho = 0.483, *p* < 0.001) (see Figure 2). Domain analyses revealed that perceived stress correlated mostly with psychological symptoms (rho = 0.503, *p* < 0.001), representing a strong correlation. Additionally, moderate correlations were observed between perceived stress and physical symptoms (rho = 0.360, *p* < 0.001) and with functional impairment (rho = 0.443, *p* < 0.001). Sleep quality and perceived stress were also weakly intercorrelated (rho = 0.235, *p* < 0.001), suggesting that poor sleep and elevated stress tend to co-occur. Additional correlational analyses with age revealed no significant correlations between age and PMS total score or domains.

To assess whether the observed associations were robust when restricted to participants with clinically meaningful symptom levels, a sensitivity analysis was conducted among only participants with moderate-to-severe PMS (total A-PMSS score > 1; n = 538, 76% of the total sample). Spearman’s rank correlation coefficients were recalculated for the three primary study variables within this subgroup. Among them, significant positive correlations persisted between sleep quality (PSQI) and PMS total score (rho = 0.278, *p* < 0.001), between perceived stress (PSS-10) and PMS total score (rho = 0.378, *p* < 0.001), and between PSQI and PSS-10 (rho = 0.235, *p* < 0.001). (Tables S2 and S3).

### 3.6. Sociodemographic and Lifestyle Variations

Non-parametric group comparisons were conducted to examine differences in PMS, sleep quality, and perceived stress across various demographic and lifestyle variables (See Table 5). The Mann–Whitney U test revealed no significant difference in PMS between medical and nursing students (*p* = 0.764), though nursing students reported significantly lower perceived stress compared to medical students (*p* = 0.007). Kruskal–Wallis test showed no significant differences in PMS across academic years (*p* = 0.231), though sleep quality differed significantly (*p* = 0.044), with fifth-year students reporting the best sleep quality (PSQI median = 5).

Additionally, caffeine consumption demonstrated significant associations with all primary variables in our study. Students reporting excessive caffeine consumption had higher PMS scores (median = 1.8) compared to those who reported less caffeine consumption (*p* = 0.014), poorer sleep quality (PSQI median = 7, *p* = 0.023), and higher stress levels (PSS median = 21, *p* = 0.021).

Moreover, physical activity level was also significantly associated with sleep quality (*p* < 0.001), with students engaging in daily physical activity reporting the best sleep quality (PSQI median = 4) but showed no significant associations with PMS (*p* = 0.504) or perceived stress (*p* = 0.470).

### 3.7. Multivariable Regression Analysis

To examine independent predictors of PMS severity, a multivariable linear regression analysis was conducted. The overall model was statistically significant (*p* < 0.001), explaining 27.5% of the variance in total PMS scores (R^2^ = 0.275; adjusted R^2^ = 0.258). Perceived stress (PSS-10 score) emerged as the strongest independent predictor of PMS severity (B = 0.047, 95% CI [0.039, 0.054], *p* < 0.001), followed by sleep quality (PSQI score: B = 0.036, 95% CI [0.024, 0.049], *p* < 0.001). Among academic year comparisons, third-year (B = 0.239, *p* = 0.015) and fifth-year students (B = 0.305, *p* = 0.016) had significantly higher PMS scores compared to first-year students. Neither caffeine consumption nor physical activity level reached statistical significance after adjustment for other variables. Variance inflation factors for all predictors were below 2.0, indicating no problematic multicollinearity. The Durbin–Watson statistic confirmed the absence of autocorrelation in the residuals (*p* = 0.1).

## 4. Discussion

This cross-sectional study investigated the relationships between sleep quality, perceived stress, and premenstrual syndrome among 708 female medical and nursing students from three major Palestinian universities. Our findings revealed that more than three-quarters (76%) of participants had moderate to severe PMS symptoms. Significant positive correlations were observed between sleep quality, perceived stress, and PMS, with perceived stress demonstrating stronger associations across all PMS domains compared to sleep quality.

Prior to this study, the relationships between sleep quality, perceived stress, and PMS had been individually examined in various populations, but their combined and independent associations among healthcare students in conflict-affected Middle Eastern settings remained largely unexplored. The present study contributes to the literature by (a) being the first to comprehensively examine the sleep–stress–PMS triad among Palestinian medical and nursing students, (b) demonstrating through multivariable regression that perceived stress (B = 0.047, *p* < 0.001) is a stronger independent predictor of PMS severity than sleep quality (B = 0.036, *p* < 0.001), together explaining 27.5% of the variance, and (c) contextualizing these findings within a conflict-affected environment where both academic and socio-political stressors converge.

The prevalence of self-reported PMS symptoms (98%) observed in our study should be interpreted as reflecting the proportion of participants endorsing any premenstrual symptom at any severity level, which differs from clinically diagnosed PMS requiring prospective confirmation. The more clinically meaningful finding is that 76% experienced moderate-to-severe symptoms, while the remaining 22% reported only mild symptoms that may represent normative premenstrual experiences. This rate is higher than the pooled estimate of 47.8% and exceeds rates reported in some previous Middle Eastern studies [36,37,38]. However, it aligns with an earlier Palestinian study documenting a 100% self-reported PMS symptom presence among Palestinian female university students [21], and another Jordanian Study reporting a 94% self-reported PMS symptom presence among premenopausal women [39].

Our finding that 62.1% of participants experienced poor sleep quality (PSQI > 5) is consistent with previous research documenting sleep disturbances among healthcare students. A systematic review and meta-analysis by Binjabr et al. [12] found that 55.64% of medical students report poor sleep quality globally. In the Arabic countries, a similar pattern has been documented, with healthcare students from Saudi Arabia, Egypt, and Yemen reporting poor sleep quality [40,41,42].

The significant association we observed between physical activity and sleep quality (*p* < 0.001), with students who exercise daily reporting the best sleep quality (PSQI median = 4), provides important insights for the role of regular physical activity. This finding aligns with research demonstrating that exercise improves sleep quality, and that could be through multiple mechanisms, including boosting melatonin production, reducing stress, and regulating body temperature, which is a key component of sleep initiation [43]. Thus, educational institutions may consider integrating structured physical activity opportunities into student schedules to support both sleep quality and overall well-being.

Our finding that 94.5% of participants experienced moderate to high perceived stress reflects the great psychological burden faced by medical and nursing students in Palestine. This prevalence is alarming, and in this Palestinian sample, it may reflect not only the academic pressures but also the broader context-specific challenges, including political instability, restricted mobility, economic constraints, and limited mental health support services. It could also be influenced by the effects of the recent conflict. Notably, data collection occurred during a period of heightened socio-political tension and active conflict in the Palestinian territories, which is likely to have amplified stress levels beyond what would be expected under routine academic conditions. The 94.5% prevalence of moderate-to-high stress likely reflects both academic pressures and the cumulative burden of living in a conflict-affected environment, including restricted mobility, economic hardship, and repeated exposure to traumatic events. Future investigations should examine how conflict-related stressors interact with academic stress to influence PMS symptom severity.

Interestingly, we found that nursing students reported significantly lower perceived stress compared to medical students (*p* = 0.007), despite no significant differences in PMS between the two groups. This finding may reflect differences in educational structure, as medical programs are often characterized by more intensive examination schedules, longer training duration, and higher competitiveness. This difference may also be related to variations in coping strategies, the strength of social support networks, or differing career expectations between the two groups. Notably, while bivariate analysis (Kruskal–Wallis) did not reveal significant differences in PMS across academic years (*p* = 0.231), the regression analysis identified significantly higher PMS severity among third-year and fifth-year students after adjusting for stress, sleep quality, and other covariates.

The weak but significant correlation we observed between sleep quality and perceived stress (rho = 0.235, *p* < 0.001) indicates that these factors tend to co-occur. The significant positive correlation we observed between sleep quality (PSQI) and total PMS scores (rho = 0.295, *p* < 0.001) aligns with Erbil and Yücesoy’s study [44], in which they reported a significant positive correlation (r = 0.311, *p* < 0.01) between sleep quality and PMS among Turkish medical and nursing students. Moreover, a systematic review revealed that women with PMS consistently reported worse subjective sleep quality, including more frequent awakenings and increased daytime fatigue [45]. This may be related to disruptions in circadian rhythm markers, particularly lower melatonin levels and elevated nocturnal core body temperature observed across the menstrual cycle [45]. Notably, these subjective complaints were not consistently reflected in objective polysomnography measures, suggesting that hormonal fluctuations may influence the perceived sleep experience without necessarily altering sleep architecture [45].

The significant bivariate associations between caffeine consumption and all three primary variables (PMS, *p* = 0.014; sleep quality, *p* = 0.023; perceived stress, *p* = 0.021) provide important insights for caffeine as a modifiable risk factor. Students reporting excessive caffeine consumption exhibited higher PMS symptom scores and poorer sleep quality compared to those who consumed less or were non-consumers. Even though caffeine intake may provide beneficial effects on alertness and cognitive performance, its consumption has also been connected to several symptoms that also occur in PMS, including depression, insomnia, headaches, and increased psychological stress [46]. In addition, a dose-dependent relationship was previously reported, as women who consume greater amounts of caffeine each day showed a higher likelihood of experiencing PMS and reported more severe symptoms; thus, reducing caffeine intake may help lessen the intensity and duration of PMS [46]. However, as per our observation, the evidence on this relationship remains inconclusive, likely due to the cross-sectional nature of most studies, the difficulty of disentangling caffeine’s effects from other lifestyle factors such as sleep deprivation and overall dietary patterns, and inconsistent definitions of “excessive” caffeine consumption across studies. In the context of healthcare education, where students often rely on caffeine to manage sleep deprivation and maintain alertness during clinical rotations, this could create a problematic cycle wherein excessive caffeine use intended to manage academic demands may sometimes worsen PMS symptoms and sleep quality.

On the other hand, the strong association of physical activity with sleep quality (*p* < 0.001), with students who exercise daily reporting PSQI scores in the good sleep range (median = 4), contrasts with the absence of significant associations between physical activity and PMS (*p* = 0.504) or perceived stress (*p* = 0.470). This pattern differs from previous research suggesting that exercise has protective effects on PMS [47,48]. However, the absence of associations with PMS and perceived stress in our study may be an artifact of the relatively low levels of physical activity in the sample, as only 10.6% engaged in regular or daily exercise. Also, the cross-sectional design may obscure potential benefits if students with more severe PMS symptoms reduce their physical activity levels.

Notably, the multivariable regression analysis revealed that neither caffeine consumption nor physical activity level reached statistical significance as independent predictors of PMS severity after adjusting for perceived stress, sleep quality, age, and academic year. This suggests that the bivariate associations observed between caffeine consumption and PMS may be partially confounded by stress and sleep quality; for example, students with higher stress and poorer sleep may consume more caffeine as a coping mechanism, rather than caffeine independently driving PMS symptoms. Similarly, the absence of a significant independent association between physical activity and PMS in the adjusted model is consistent with the low levels of physical activity in this sample and warrants further investigation in populations with greater activity variability.

### 4.1. Study Implications

The findings of our study have several important implications for healthcare education and student support services. The high prevalence of moderate to severe PMS (76%), coupled with the high prevalence of poor sleep quality (62.1%) and elevated moderate to severe stress (94.5%), indicates substantial unmet health needs among female medical and nursing students in Palestine. Universities should prioritize the development and implementation of comprehensive wellness programs addressing these interconnected issues. Stress management training should incorporate cognitive–behavioral techniques, mindfulness-based approaches, and time management skills to help students develop effective coping strategies. Sleep hygiene education is also essential to address behavioral factors that impair sleep quality and promote restorative rest. Menstrual health education should normalize PMS experiences and provide evidence-based management strategies, empowering students with knowledge about their reproductive health. Universities could also integrate stress management and sleep hygiene modules into existing orientation programs or clinical skills courses. Additionally, peer-support groups could serve as a cost-effective mechanism for menstrual health education and normalization of these experiences. Finally, training academic advisors and clinical supervisors to recognize signs of stress, poor sleep, and PMS-related difficulties could facilitate early intervention and appropriate referral to support services.

### 4.2. Strengths and Limitations

Our study’s strengths include its relatively large sample size (N = 708), recruitment from three major Palestinian universities, enhancing generalizability within the Palestinian healthcare education context, the use of validated Arabic instruments, the comprehensive assessment of PMS across psychological, physical, and functional domains, and the appropriate application of non-parametric statistical methods for non-normally distributed data.

However, several limitations warrant consideration. First, our cross-sectional design precludes causal inference and limits the ability to establish temporal relationships between sleep quality, perceived stress, and PMS. Second, our reliance on self-report introduces potential recall bias. Third, our study did not include objective measures of sleep quality (e.g., polysomnography) or biological markers of stress (e.g., cortisol, inflammatory cytokines) that could clarify physiological mechanisms underlying observed associations. Fourth, our use of convenience sampling via social media, while necessary given the contextual constraints in the Palestinian setting, may introduce self-selection bias. Specifically, students experiencing more severe PMS symptoms, higher stress levels, or poorer sleep quality may have been disproportionately motivated to participate in a study focused on these topics, potentially leading to overestimation of prevalence rates. Additionally, because the survey was distributed online, a precise response rate cannot be calculated. Fifth, the A-PMSS relies on retrospective self-report of symptoms, without prospective daily symptom tracking. Retrospective reporting is susceptible to recall bias and may inflate prevalence estimates compared to prospective diary-based assessment, which is considered the gold standard for PMS diagnosis. Sixth, our study assessed self-reported PMS symptom presence using a validated screening tool, which does not constitute a formal clinical diagnosis of PMS. Our prevalence figure of 98% reflects any symptom endorsement at any severity level, with the moderate-to-severe subgroup (76%) more closely approximating clinically relevant PMS. Readers should interpret these findings accordingly.

### 4.3. Future Research Directions

Future research may benefit from employing prospective longitudinal designs following students across multiple menstrual cycles to clarify temporal relationships between sleep quality, perceived stress, and PMS. In addition, mixed-methods research incorporating qualitative approaches would enhance our understanding of the lived experiences of PMS among students, barriers to seeking help, coping strategies employed, and preferences for intervention delivery formats. Such research could inform culturally appropriate, contextually relevant interventions that align with students’ needs and values. Future research should also focus on developing and testing culturally sensitive interventions, such as cognitive–behavioral therapy for insomnia (CBT-I) adapted for student populations, structured physical activity programs, and mindfulness-based stress reduction approaches, to determine their efficacy in improving sleep, reducing stress, and alleviating PMS symptoms.

## 5. Conclusions

This study reveals a substantial burden of self-reported premenstrual symptoms, poor sleep quality, and elevated perceived stress among female medical and nursing students in Palestine. Nearly all participants (98%) reported some degree of PMS symptoms, with 76% experiencing moderate-to-severe symptoms; 62.1% demonstrated poor sleep quality, and 94.5% reported moderate-to-high perceived stress. Multivariable regression analysis demonstrated that perceived stress and sleep quality are independently associated with PMS symptom severity, together explaining 27.5% of the variance, with perceived stress emerging as the stronger predictor. These findings underscore the need for Palestinian universities and healthcare education institutions to implement comprehensive wellness programs integrating stress management training, sleep hygiene education, and menstrual health support. Such interventions should be culturally appropriate, contextually relevant, and tailored to address the unique challenges faced by students living in a conflict-affected environment.

## Figures and Tables

**Table 1:** Sociodemographic Characteristics of Participants (n = 708).

Characteristic	Median (IQR) or n (%)
Age (Years)	21 (3)
Marital Status	
Single	686 (96.9%)
Married	21 (3%)
Divorced	1 (0.1%)
Living Arrangement	
With family/parents	520 (73.4%)
University dorms	136 (19.2%)
Shared apartment with roommates	21 (3%)
Alone	12 (1.7%)
With spouse	19 (2.7%)
Residence	
City	396 (56%)
Village	292 (41.2%)
Camp	20 (2.8%)
University	
Al-Quds University	214 (30.2%)
An-Najah National University	317 (44.8%)
Hebron University	177 (25%)
Major	
Medicine	419 (59.2%)
Nursing	289 (40.8%)
Academic Year	
First Year	88 (12.4%)
Second Year	126 (17.8%)
Third Year	108 (15.3%)
Fourth Year	202 (28.5%)
Fifth Year (Medicine)	137 (19.4%)
Sixth Year (Medicine)	47 (6.6%)

Note: Data presented as median (IQR) for continuous variables and n (%) for categorical variables.

**Table 2:** Clinical and Lifestyle Characteristics of Participants (n = 708).

Characteristic	Median (IQR) or n (%)
Menarche Age (Years)	13 (2)
Menstrual Pain Severity	
No Pain	30 (4.2%)
Mild (does not affect daily activities)	188 (26.6%)
Moderate (sometimes affects daily activities)	313 (44.2%)
Severe (frequently affects daily activities)	177 (25%)
Caffeine Consumption	
Never	52 (7.3%)
Rarely (1–2 times per week)	126 (17.8%)
Sometimes (3–4 times per week)	160 (22.6%)
Daily (1–2 cups/glasses per day)	301 (42.5%)
Excessive consumption (more than 3 cups/glasses per day)	69 (9.7%)
Physical Activity Level	
Never	164 (23.2%)
Rarely (less than once per week)	263 (37.1%)
Sometimes (1–2 times per week)	206 (29.1%)
Regularly (3–4 times per week)	53 (7.5%)
Daily or almost daily	22 (3.1%)
Smoking	
Never	671 (94.8%)
Former smoker	13 (1.8%)
Occasional smoker (less than 5 cigarettes per week)	20 (2.8%)
Regular smoker (5 cigarettes or more per week)	4 (0.6%)

Note: Data presented as median (IQR) for continuous variables and n (%) for categorical variables.

**Table 3:** Prevalence, Severity, and Summary Statistics for PMS, Sleep Quality, and Perceived Stress (n = 708).

Variable	n	%	Median (IQR)	Mean (SD)
PMS Severity (A-PMSS)				
None (score = 0)	14	2.0	-	-
Mild (0 < score ≤ 1)	156	22.0	-	-
Moderate (1 < score ≤ 2)	333	47.0	-	-
Severe (2 < score ≤ 3)	205	29.0	-	-
Moderate to Severe Combined	538	76.0	-	-
Total PMS Score	-	-	1.60 (1.04)	1.58 (0.68)
Psychological Domain	-	-	1.75 (1.17)	1.70 (0.77)
Physical Domain	-	-	1.50 (1.00)	1.53 (0.68)
Functional Impairment	-	-	1.33 (1.33)	1.30 (0.86)
Sleep Quality (PSQI)				
Good Sleep (PSQI ≤ 5)	268	37.9	-	-
Poor Sleep (PSQI > 5)	440	62.1	-	-
Global PSQI Score	-	-	7 (5)	7.11 (3.62)
Perceived Stress (PSS-10)				
Low Stress (0–13)	39	5.5	-	-
Moderate Stress (14–26)	516	72.9	-	-
High Stress (27–40)	153	21.6	-	-
Moderate to High Combined	669	94.5	-	-
PSS-10 Score	-	-	20.5 (9)	21.7 (6.18)

Note: PMS = Premenstrual Syndrome (A-PMSS; scores 0–3; higher = more severe); PSQI = Pittsburgh Sleep Quality Index (scores 0–21; higher = poorer sleep quality; >5 = poor sleep); PSS-10 = Perceived Stress Scale (scores 0–40; higher = greater stress). IQR = interquartile range; SD = standard deviation.

**Table 4:** Spearman’s Rank Correlation Coefficients Between Sleep Quality, Perceived Stress, and PMS Variables (n = 708).

Variables	Spearman’s rho	*p*-Value
Sleep Quality (PSQI) and PMS Symptoms		
PSQI & Total PMS Score	0.295	<0.001
PSQI & Psychological Symptoms	0.248	<0.001
PSQI & Physical Symptoms	0.278	<0.001
PSQI & Functional Impairment	0.317	<0.001
Perceived Stress (PSS-10) and PMS Symptoms		
PSS & Total PMS Score	0.483	<0.001
PSS & Psychological Symptoms	0.503	<0.001
PSS & Physical Symptoms	0.360	<0.001
PSS & Functional Impairment	0.443	<0.001
Other Correlations		
PSQI & PSS	0.235	<0.001
Age & Total PMS	0.034	0.362
Age & psychological symptoms	0.036	0.344
Age & physical symptoms	0.017	0.661
Age & functional impairment	0.057	0.131

Note: PSQI = Pittsburgh Sleep Quality Index; PSS = Perceived Stress Scale; PMS = Premenstrual Syndrome.

**Table 5:** Comparison of PMS, Sleep Quality (PSQI), and Perceived Stress (PSS-10) Scores Across Sociodemographic and Lifestyle Variables Using Non-Parametric Tests (N = 708).

Variable	Total PMS Score Median (IQR)	PSQI Score Median (IQR)	PSS Score Median (IQR)
Major			
Medicine (n = 419)	1.6 (1.12)	6 (5)	21 (9)
Nursing (n = 289)	1.56 (0.92)	7 (6)	20 (7)
Mann–Whitney U test	*p*-value = 0.764	*p*-value = 0.189	*p*-value = 0.007
Academic Year			
First Year (n = 88)	1.48 (1.04)	6 (4)	21 (8.25)
Second Year (n = 126)	1.50 (1.07)	7 (6)	20 (8)
Third Year (n = 108)	1.78 (0.93)	7 (5)	21 (7.25)
Fourth Year (n = 202)	1.64 (0.96)	7 (5)	20 (9)
Fifth Year (n = 137)	1.68 (1.08)	5 (4)	20 (9)
Sixth Year (n = 47)	1.56 (1.12)	7 (4)	21 (7.5)
Kruskal–Wallis test	*p*-value = 0.231	*p*-value = 0.044	*p*-value = 0.510
Caffeine Consumption			
Never (n = 52)	1.34 (1.2)	5 (3)	19 (6.25)
Rarely (n = 126)	1.48 (1.03)	6 (5)	20 (8)
Sometimes (n = 160)	1.56 (0.97)	6 (5)	20 (7)
Daily (n = 301)	1.68 (1.04)	7 (4)	21 (9)
Excessive consumption (n = 69)	1.80 (0.92)	7 (6)	21 (12)
Kruskal–Wallis test	*p*-value = 0.014	*p*-value = 0.023	*p*-value = 0.021
Physical Activity Level			
Never (n = 164)	1.68 (1.08)	6 (6)	21 (9)
Rarely (n = 263)	1.56 (1.02)	7 (5)	21 (9)
Sometimes (n = 206)	1.56 (0.99)	7 (5)	20 (8)
Regularly (n = 53)	1.72 (0.8)	5 (4)	21 (9)
Daily or almost daily (n = 22)	1.64 (1.49)	4 (3)	21 (7.75)
Kruskal–Wallis test	*p*-value = 0.504	*p*-value < 0.001	*p*-value = 0.470

Note: PMS = Premenstrual Syndrome (total score ranges 0–3); PSQI = Pittsburgh Sleep Quality Index (scores range 0–21); PSS = Perceived Stress Scale (scores range 0–40). Higher scores indicate greater severity for all variables.

## Data Availability

The data presented in this study are available on request from the corresponding author due to privacy restrictions.

## Supplementary Materials

The following supporting information can be downloaded at: https://www.mdpi.com/article/10.3390/healthcare14091168/s1, Table S1: STROBE Checklist; Table S2: Spearman’s rank correlation coefficients among participants with moderate to severe PMS; Table S3: Comparison of Spearman’s correlations: full sample vs. moderate to severe PMS Subgroup.
