# Supplementary material for: Relationships Between Sleep Quality, Perceived Stress, and Premenstrual Syndrome Among Medical and Nursing Students: A Cross-Sectional Study in Palestine
Source: Healthcare (Basel). 2026 Apr 27;14(9):1168. doi: 10.3390/healthcare14091168 (PMC13163972; doi:10.3390/healthcare14091168)
Supplement: Supplementary file 1 [file healthcare-14-01168-s001.zip › healthcare-4166952-supplementary.pdf]

Supplementary Materials

Table S1. STROBE Statement—Checklist of items that should be included in reports of ***cross-sectional studies***.

	**Item No**	**Recommendation**	**Page No**
**Title and abstract**	1	(*a*) Indicate the study's design with a commonly used term in the title or the abstract	1
		(*b*) Provide in the abstract an informative and balanced summary of what was done and what was found	2-3
		Introduction	
Background/rationale	2	Explain the scientific background and rationale for the investigation being reported	4-6
Objectives	3	State specific objectives, including any prespecified hypotheses	7
		Methods	
Study design	4	Present key elements of study design early in the paper	8
Setting	5	Describe the setting, locations, and relevant dates, including periods of recruitment, exposure, follow-up, and data collection	8
Participants	6	(*a*) Give the eligibility criteria, and the sources and methods of selection of participants	8
Variables	7	Clearly define all outcomes, exposures, predictors, potential confounders, and effect modifiers. Give diagnostic criteria, if applicable	9-11
Data sources/ measurement	8*	For each variable of interest, give sources of data and details of methods of assessment (measurement). Describe comparability of assessment methods if there is more than one group	*9-11*
Bias	9	Describe any efforts to address potential sources of bias	--
Study size	10	Explain how the study size was arrived at	9
Quantitative variables	11	Explain how quantitative variables were handled in the analyses. If applicable, describe which groupings were chosen and why	11-12
Statistical methods	12	(*a*) Describe all statistical methods, including those used to control for confounding	11-12
		(*b*) Describe any methods used to examine subgroups and interactions	12
		(*c*) Explain how missing data were addressed	12
		(*d*) If applicable, describe analytical methods taking account of sampling strategy	--
		(*e*) Describe any sensitivity analyses	Supp 2
		Results	
Participants	13*	(a) Report numbers of individuals at each stage of study—e.g., numbers potentially eligible, examined for eligibility, confirmed eligible, included in the study, completing follow-up, and analysed	13
		(b) Give reasons for non-participation at each stage	--
		(c) Consider use of a flow diagram	--
Descriptive data	14*	(a) Give characteristics of study participants (e.g., demographic, clinical, social) and information on exposures and potential confounders	13-16
		(b) Indicate number of participants with missing data for each variable of interest	--
Outcome data	15*	Report numbers of outcome events or summary measures	17
Main results	16	(*a*) Give unadjusted estimates and, if applicable, confounder-adjusted estimates and their precision (e.g., 95% confidence interval). Make clear which confounders were adjusted for and why they were included	23

		(*b*) Report category boundaries when continuous variables were categorized	17
		(*c*) If relevant, consider translating estimates of relative risk into absolute risk for a meaningful time period	--
Other analyses	17	Report other analyses done—e.g., analyses of subgroups and interactions, and sensitivity analyses	21-22 Supp file 2
		Discussion	
Key results	18	Summarise key results with reference to study objectives	24
Limitations	19	Discuss limitations of the study, taking into account sources of potential bias or imprecision. Discuss both direction and magnitude of any potential bias	30
Interpretation	20	Give a cautious overall interpretation of results considering objectives, limitations, multiplicity of analyses, results from similar studies, and other relevant evidence	25-28
Generalisability	21	Discuss the generalisability (external validity) of the study results	29
		Other information	
Funding	22	Give the source of funding and the role of the funders for the present study and, if applicable, for the original study on which the present article is based	---

*Give information separately for exposed and unexposed groups.

Note: An Explanation and Elaboration article discusses each checklist item and gives methodological background and published examples of transparent reporting. The STROBE checklist is best used in conjunction with this article (freely available on the Web sites of PLoS Medicine at http://www.plosmedicine.org/, Annals of Internal Medicine at http://www.annals.org/, and Epidemiology at http://www.epidem.com/). Information on the STROBE Initiative is available at www.strobe-statement.org.

To assess whether the observed associations were robust when restricted to participants with clinically meaningful symptom levels, a sensitivity analysis was conducted among only participants with moderate to severe PMS (total A-PMSS score > 1; n = 538, 76% of the total sample). Spearman's rank correlation coefficients were recalculated for the three primary study variables within this subgroup (Table S2).

Table S2. Spearman's rank correlation coefficients among participants with moderate to severe PMS (n = 538).

Variables	Spearman's rho	p-value
	Sleep Quality (PSQI) & PMS	
PSQI & Total PMS Score	0.278	<0.001
	Perceived Stress (PSS-10) & PMS	
PSS & Total PMS Score	0.378	<0.001
	Sleep Quality (PSQI) &Perceived Stress (PSS-10)	
PSQI & PSS-10	0.235	<0.001

Among participants with moderate to severe PMS, significant positive correlations persisted between sleep quality (PSQI) and PMS total score (rho = 0.278, $p < 0.001$), between perceived stress (PSS-10) and PMS total score (rho = 0.378, $p < 0.001$), and between PSQI and PSS-10 (rho = 0.235, $p < .001$). As expected, the magnitude of the PSS-PMS correlation was attenuated compared to the full sample (rho = 0.378 vs. 0.483), reflecting the reduced variance in PMS scores when the analysis is restricted to a narrower severity range. Similarly, the PSQI-PMS correlation showed minimal attenuation (rho = 0.278 vs. 0.295).

Importantly, all associations remained statistically significant at $p < .001$, confirming that the observed relationships are not driven by the inclusion of participants with mild or normative premenstrual symptoms and are robust when restricted to clinically meaningful symptom levels. The stability of the PSQI-PSS correlation (rho = 0.235 in both the full sample and the subgroup) further indicates that the co-occurrence of poor sleep and elevated stress is consistent across the PMS severity spectrum and is not an artefact of including participants with minimal symptoms (Table S3).

Table S3. Comparison of spearman's correlations: full sample vs. moderate to severe PMS subgroup.

Variable Pair	Full Sample (N = 708)	Moderate to Severe (n = 538)	Δ rho
PSQI & Total PMS Score	0.295	0.278	-0.017
PSS & Total PMS Score	0.483	0.378	-0.105
PSQI & PSS-10	0.235	0.235	0

Note: Δ rho = change in correlation coefficient from the full sample to the moderate-to-severe subgroup. Negative values indicate attenuation, which is expected due to restriction of range in PMS scores. All correlations remained significant at $p < .001$ in both the full sample and the subgroup.
